# Attitudes and confidence toward deprescribing: a survey among Italian general practitioners

**DOI:** 10.1186/s12875-025-03042-2

**Published:** 2025-12-23

**Authors:** Andrea Rossi, Stefano Scotti, Lara Perrella, Federica Galimberti, Elena Olmastroni, Enrica Menditto, Valentina Orlando, Ilaria Ardoino, Carlotta Franchi, Manuela Casula

**Affiliations:** 1 Epidemiology and Preventive Pharmacology Service (SEFAP), Department of Pharmacological and Biomolecular Sciences, University of Milan, Milan, Italy; 2 IRCCS MultiMedica, Sesto San Giovanni, Milan, Italy; 3 Center of Pharmacoeconomics and Drug Utilization Research, Department of Pharmacy, CIRFF, University of Naples Federico II, Naples, Italy; 4 Laboratory of Pharmacoepidemiology and Human Nutrition, Department of Health Policy, Istituto di Ricerche Farmacologiche Mario Negri IRCCS, Milan, Italy

**Keywords:** Deprescribing, General practice, Survey, Medication review, Polypharmacy

## Abstract

**Background:**

General practitioners (GPs) should regularly review patients’ medications and, when they are potentially harmful or no longer necessary, implement deprescribing approach. We aimed to assess the perceptions and potential barriers to deprescribing among Italian GPs.

**Methods:**

GPs were invited to participate in an observational cross-sectional study through an online survey containing 20 questions addressing attitudes towards deprescribing, including physicians’ perceptions, potential barriers, and how this process is addressed in daily clinical practice. The survey, accessible for responses from 4th May 2023 to 15th January 2024, was distributed through social media, networks, medical associations, and involving primary care departments of local health authorities.

**Results:**

Over 8 months, 617 answers were collected. Less than 2% (*n* = 11) reported to not implement deprescribing interventions in daily practice, primarily due to perceived insufficient experience (*n* = 7) or lack of specific education (*n* = 6). Conversely, 23.1%(*n* = 142) of respondents reported frequently or very frequently implementing deprescribing. Among barriers, GPs reported difficulties in dealing with specialists (*n* = 438, 71.7%), distrust of patients in drug discontinuation (*n* = 326, 53.4%), poor availability of deprescribing guidelines (*n* = 231, 37.8%), and time constraints (*n* = 213, 34.9%). Guidelines and targeted training were mostly demanded (by 66.1% and 59.6%, respectively). Regarding specific drug classes, the proportion of GPs reporting to frequently implement deprescribing for proton pump inhibitors was 51.8% (*n* = 313), while percentage was lower for benzodiazepines (*n* = 166, 27.4%), bisphosphonates (*n* = 147, 24.3%), statins (*n* = 128, 21.2%), antihypertensives (*n* = 108, 17.9%), and antidepressants (*n* = 96, 15.9%).

**Conclusion:**

The study shows that while GPs recognize the importance of deprescribing, however, they face significant barriers, including a lack in targeted education and specific guidelines to enhance their confidence and knowledge in implementing this process effectively in daily clinical practice.

**Supplementary Information:**

The online version contains supplementary material available at 10.1186/s12875-025-03042-2.

## Introduction

Pharmacological therapies play a crucial role in managing a wide range of health conditions, improving patient outcomes, and enhancing quality of life. However, not all prescribed medications remain necessary or appropriate over time [1]. Some drugs may be initially justified but become less beneficial or even harmful as a patient’s clinical condition changes. For example, medications may pose increased risks due to age-related physiological changes, including decreased renal and hepatic function, or to drug-drug interactions. In such cases, continuing the same treatments without periodic reassessment can contribute to adverse outcomes, such as falls, cognitive impairment, or hospitalizations [2].

Healthcare practitioners and researchers have many tools available to combat this ever-growing problem [3, 4], one of them being deprescribing [5]. Deprescribing is a systematic process aimed at reviewing, identifying, modifying, and discontinuing medications when the risks of adverse effects outweigh the benefits. It is a patient-centered approach that considers the individual’s health goals, current functional status, life expectancy, personal values, and preferences [6, 7].

The practice of deprescribing has gained increasing attention as a way to improve health outcomes, reduce the burden of polypharmacy, and enhance overall quality of life in older adults [8–10]. Recent studies show that deprescribing can reduce adverse drug reactions by 30% and improve medication adherence by 20%, leading to better clinical outcomes [11–13], such as a significant reduction in falls [14] or in hospitalizations [15]. By carefully balancing the need for medication with the potential for harm, deprescribing can play a crucial role in optimizing care for this vulnerable population [16, 17].

In many countries, as in Italy, the general practitioner (GP) plays a pivotal role in the healthcare system, acting as the first point of contact for most patients and serving as the gatekeeper to specialist care. All citizens are registered with a GP as part of the state-funded National Health Service (NHS), making GPs central to the management of chronic conditions, including the prescription and monitoring of long-term medications. This unique position places GPs at the forefront of patient care, providing them with a comprehensive understanding of the patient’s medical history, current treatments, and overall health, enabling them to effectively assess the necessity of continued medication use [18, 19].

One of the key responsibilities of GPs in managing long-term therapies is conducting regular medication reviews. These reviews provide an opportunity to assess the ongoing appropriateness of each drug, consider potential drug-drug interactions, and evaluate the patients’ current health status in relation to their prescribed treatments. Several tools support GPs in identifying Potentially Inappropriate Medications (PIMs) and guiding deprescribing. The Beers Criteria, START/STOPP, FORTA and PRISCUS lists provide evidence-based frameworks to optimize prescribing and reduce polypharmacy-related risks [20–23]. By performing regular assessments, GPs can identify medications that may no longer be necessary and initiate the deprescribing process where appropriate, considering not only the clinical indications for deprescribing but also patient’s preferences, quality of life, and potential side effects from polypharmacy [24, 25]. Several countries have introduced collaborative care models involving GPs, pharmacists, and sometimes nurses to improve medication management and address workload challenges. These models, such as medication reviews in Australia and Canada [26], focus on optimizing prescriptions through reconciliation, patient-specific evaluations, and interaction assessments. Pharmacists also provide education and counselling, and physicians often accept their recommendations for medication adjustments or deprescribing. In countries like the UK and Slovenia, the involvement of clinical pharmacists shows important results in prescribing, deprescribing, and optimizing medication therapy, reducing polypharmacy, improve guideline adherence, and decrease PIMs [27–29].

Despite the ideal position of GPs, and the health benefits associated with this approach, deprescribing is not always systematically integrated into clinical practice [30]. Some studies have suggested potential barriers and obstacles to the full implementation of deprescribing in primary care [24, 31]. Adding the point of views of GPs to this evidence could provide the basis for ameliorating deprescribing implementation in clinical practice. The aim of this survey was to explore the GPs’ experiences with deprescribing, identify specific areas where additional support is needed, and pinpoint the main barriers encountered or perceived in daily practice. A particular focus of the survey was placed on proton pump inhibitors (PPIs), a class of drugs often prescribed inappropriately in both the general and older populations [32].

## Methods

### Study design

A cross-sectional survey was conducted to assess GPs’ attitudes and confidence towards deprescribing practices. The on-line survey comprised 20 structured questions and was designed to cover a range of topics related to deprescribing. The questionnaire was specifically developed for this study (**Supplementary material**), based on existing literature, expert input, and guidelines related to deprescribing in primary care settings [33]. The Italian version used in this survey was reviewed by colleagues from various fields to ensure clarity and comprehensibility.

The questions were formulated to capture GPs’ attitudes toward deprescribing, perceived challenges they face in implementing it, and their confidence in discussing this topic with patients. The survey was divided into the following main sections:

i) Demographics: this section collected general information about the respondents, such as gender, years of clinical experience (less than 5 years of experience, between 5 and 25 years of experience, and over 25 years of experience), and the geographical region where they practice (Northern, Central or Southern Italy). This was important to explore potential differences in deprescribing attitudes based on these factors.
ii) Attitudes towards deprescribing: questions in this section aimed to gauge GPs’ perspectives on the importance and relevance of deprescribing approach. Items included statements about their agreement or disagreement with deprescribing for specific patient groups, and their perception of the risks and benefits.
iii) Confidence in deprescribing: this section measured how confident GPs felt about initiating deprescribing, discussing it with patients, and identifying appropriate candidates for deprescribing.
iv) Barriers to deprescribing: this section includes questions addressing common barriers, such as time constraints, concerns about patient resistance, lack of guidelines, or fear of adverse outcomes due to treatment discontinuation.
v) Deprescribing in clinical practice: GPs were asked about how often they engage in deprescribing, the types of medications they typically deprescribe, with a particular focus on PPIs.

The specific focus on PPIs stems from the context in which this survey was developed, namely the LAPTOP-PPI randomized controlled trial [32, 34], which aimed to improve the appropriateness of PPI prescribing among Italian GPs. This emphasis reflects national data showing the significant financial impact of PPIs on the healthcare system: pantoprazole ranked as the second highest active ingredient in terms of expenditure, and four of the top ten most costly drugs were PPIs. These findings underscore the substantial burden PPIs place on the NHS, justifying the inclusion of a dedicated section in the survey specifically addressing PPI deprescribing.

### Survey distribution

The survey was distributed through various channels to ensure broad participation. These included social media platforms, mailing lists of scientific societies and platforms, and professional networks. Additionally, the survey was shared via local and national medical societies, particularly those with a focus on general practice and primary care. Collaborations were established with the primary care departments of local health authorities to further help disseminate the survey and reach a wide audience of GPs. To maximize the response rate, reminders were sent through the same distribution channels after the initial survey distribution. This approach, aimed at maximizing the number of GPs reached by the survey, prevents us from providing an estimate of how many GPs actually received the questionnaire, and therefore from calculating a response rate. Participation was voluntary, and no incentives were provided. The on-line survey was available from the 4th of May 2023 to 15th of January 2024.

### Ethical considerations

Since the questionnaires did not concern health data or sensitive information but rather surveys on the general approach and opinions of clinicians regarding deprescribing, it was not necessary to request approval from an ethics committee, according to the Italian Data Protection Code (Legislative Decree30 June 2003, n. 196) and the provisions of Regulation (EU) 2016/679 (GDPR). Before participating, GPs were informed about the purpose of the study, and consent was obtained via an introductory page in the survey (by submitting the questionnaire, the GP confirmed having read and understood the data management notice; those who did not agree could simply discontinue the completion of the questionnaire). Participation was totally anonymous, and no personally identifiable information was collected. All data were stored securely, and only aggregated results were reported.

### Data collection and analysis

The survey responses were collected electronically and stored in a secure database. All data were exported and analyzed through Statistical Package for Social Science (SPSS) version 29.0 (IBM SPSS, Armonk, NY: IBM Corp.). Descriptive statistics were used to summarize the demographic characteristics of the respondents and their responses to each survey item. Frequency distributions and percentages were calculated for categorical variables. Where appropriate, stratified analyses were conducted to explore associations between GPs’ attitudes toward deprescribing and years of experience.

## Results

### Demographic characteristics of respondents

Over a period of eight months, a total of 617 responses were collected from GPs across the country, providing a comprehensive dataset representative of the national landscape. The GP cohort was notably gender-balanced, with 50.1% (*n* = 309) of respondents identifying as male and 49.4% (*n* = 305) as female, while 0.5% (*n* = 3) chose not to specify their gender. In terms of clinical experience, 22.9% (*n* = 141) reported having less than 5 years of professional experience, 32.2% (*n* = 199) had between 5 and 25 years, while 44.9% (*n* = 277) had over 25 years.

### Perceptions of deprescribing

Only 3.6% (*n* = 22) of the respondents considered deprescribing to be slightly relevant or irrelevant; 35.4% (*n* = 218) considered deprescribing quite relevant and 41.7% (*n* = 257) considered it very relevant. The latter percentage decreased with increasing years of experience, from 46.8% in GPs with less than 5 years of clinical experience to 36.5% in more experienced ones.

**Fig. 1:**
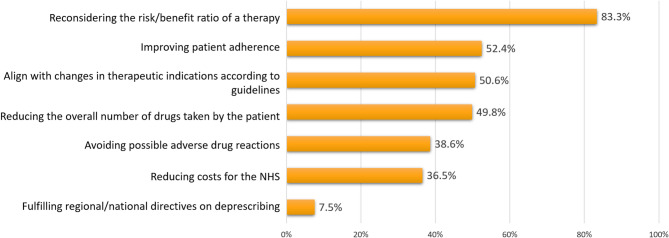
Responses to the question “Why a doctor should implement deprescribing” (respondents could provide more than one answer)

Answering the question about why deprescribing should be implemented, 83.3% (*n* = 509) of responders recognized the need of a risk/benefit re-evaluation for a treatment. About half of the sample indicated the purpose of improving patient adherence, aligning with recent guidelines, and reducing the overall numbers of treatments in a patient (Fig. 1). Interestingly, the purpose of reducing NHS costs was reported by 56.4% of less experienced GPs, but only by 24.2% of GPs with more than 25 years of experience (Supplementary Fig. 1).

### Role of GPs in deprescribing

**Fig. 2:**
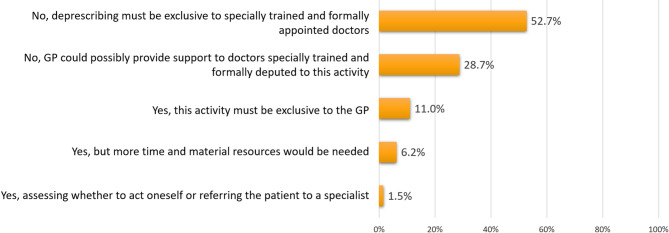
Responses to the question “Do you agree with the statement that deprescribing should be in charge to the general practitioner?” (respondents could provide only one answer)

Regarding the professional figure responsible for the deprescribing process, 52.7% (*n* = 325) of the respondents believed it should not be entrusted to GPs but rather reserved for professionals specifically trained and formally designated for this task. In contrast, 28.7% (*n* = 177) supported the idea of GPs playing a supportive role alongside specialists (Fig. 2). The exclusive role of the GP in the deprescribing practice, indicated overall by 11.0% (*n* = 65) of the sample, was reported by 17.0% of GPs with more years of experience and by 2.1% of less experienced GPs (Supplementary Fig. 2).

### Implementation of deprescribing in GP practice

When questioned about the implementation of deprescribing in daily practice, 11 GPs (1.8%) reported not implementing interventions routinely, primarily due to perceived insufficient experience (*n* = 7) and lack of specific education (*n* = 6). Conversely, 23.1%(*n* = 142) of respondents reported frequently or very frequently implementing deprescribing (ranging from 19.1% in GPs with less than 5 years of experience to 24.6% in those with 25 years of experience or more). Most of the interviewed GPs indicated a more frequently implementation of deprescribing in very older patients (over 80 years, 58.8%) or older patients (over 64 years, 30.7%), while deprescribing in patients aged 64 or less was reported by only 10.4% of the sample. The main triggers for initiating deprescribing were reported to be adverse drug effects and the presence of comorbidities/polypharmacy, indicated by 77.6% (*n* = 479) and 75% (*n* = 463) of respondents, respectively.

### Barriers to deprescribing

**Fig. 3:**
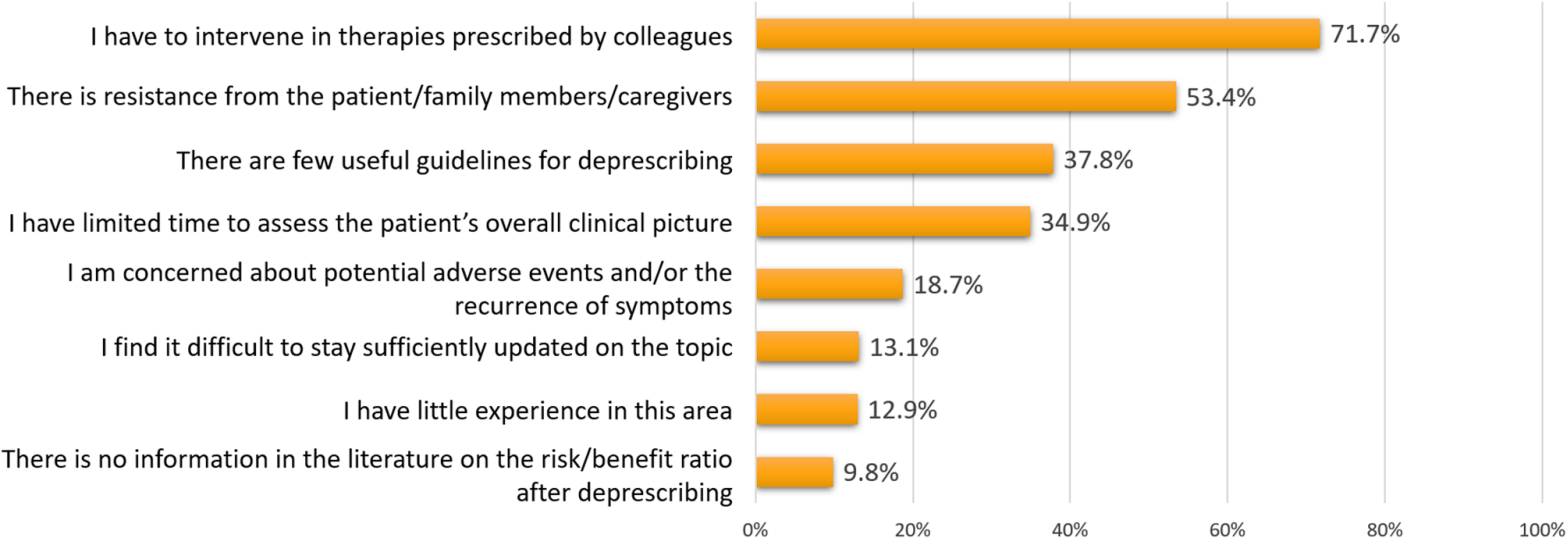
Responses to the question “What do you perceive as the main obstacles or challenges in the deprescribing process?” (respondents could provide more than one answer)

When asked about perceived barriers to implementing deprescribing, 71.7% (*n* = 438) of respondents indicated the need to intervene in therapies prescribed by colleagues or other specialists (Fig. 3)and time constraints (*n* = 213, 34.9%).

Resistance from patients or caregivers was reported by 53.8% (*n* = 326), while about one in three respondents mentioned the lack of guidelines and limited time availability to comprehensively assess the patient’s clinical condition as obstacles (*n* = 231, 37.8%). Notably, the latter response showed the greatest variability based on years of professional experience, being reported by 50.4% of GPs with less than 5 years of experience and by 23.8% of those with 25 years of experience or more (Supplementary Fig. 3).

### Tools to support deprescribing

**Fig. 4:**
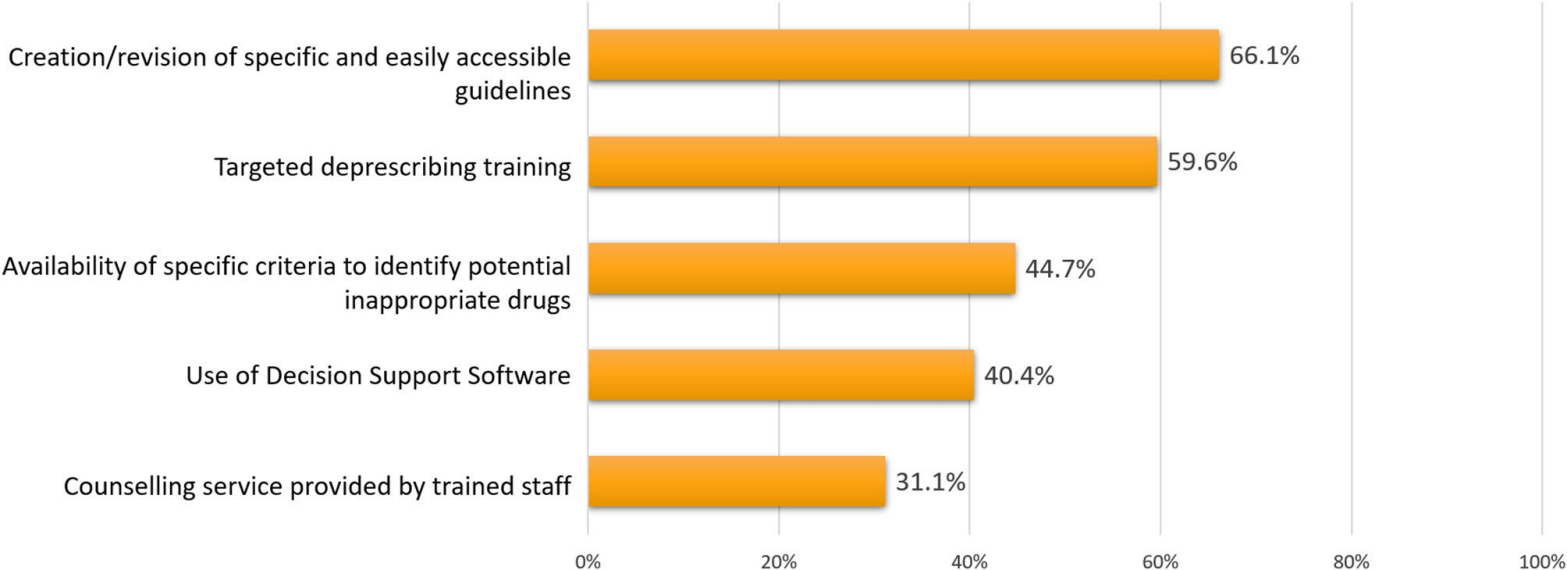
Responses to the question “Which of the following instruments could most facilitate deprescribing?” (respondents could provide more than one answer)

When GPs were asked to indicate which tools could facilitate the implementation of deprescribing, 66.1% (*n* = 408) of respondents mentioned specific and easily accessible guidelines (Fig. 4), followed by targeted training, (*n* = 368, 59.6%). Access to consultation services was selected by only 31.1% (*n* = 192) of the sample. Stratification by years of experience revealed significant differences, particularly regarding access to specific criteria (cited by 57.4% of GPs with less than 5 years of experience but only 37.2% of those with over 25 years of experience) and the availability of decision-support software (indicated by 56.0% and 32.5%, respectively) (Supplementary Fig. 4).

### Deprescribing of specific drug classes

GPs were then asked to indicate how frequently certain drug classes are deprescribed, specifically benzodiazepines, antidepressants, antihypertensives, statins, bisphosphonates, and proton pump inhibitors (PPIs). Deprescribing antidepressants and antihypertensives was a common practice for less than one in five GPs, 15.9% (*n* = 96) and 17.9% (*n* = 108), respectively. Notably, there was significant variability in antidepressant deprescribing based on years of professional experience, with less experienced GPs reporting routinely deprescribing in only 6.0% of cases, compared to 23.4% of those with 25 or more years of experience. Routinely deprescribing of statins, bisphosphonates, and benzodiazepines was reported by 21.2% (*n* = 128), 24.3% (*n* = 147), and 27.4% (*n* = 166) of respondents, respectively, with no significant variations based on years of experience. Regarding PPIs, their deprescribing was reported to be frequently performed by 51.8% (*n* = 313) of the interviewed GPs, ranging from 46.1% among those with at least 25 years of experience to 56.8% among those with less than 5 years of experience. Specifically, for this class of drugs, the most frequently reported reasons for initiating deprescribing were: (i) the therapy was no longer necessary (81.7%) and (ii) the clinical issue could be managed through lifestyle interventions (69.9%). Patient acceptability of PPI deprescribing was reported as unsatisfactory by 39.1% of respondents.

## Discussion

This survey shows that the vast majority of the interviewed doctors agree on the importance of deprescribing, but this practice is only occasionally implemented in daily clinical practice. This data, related to Italian general medicine, confirms an already well-described evidence in the literature. A survey conducted in France in 2016 [35], nearly all respondents felt comfortable or fairly comfortable in deprescribing inappropriate medications, but only 35% decided to do so often or very often. In the recent study by Coelho et al. [36] conducted in Portugal, deprescribing was reported as a frequent practice by 32% of the 396 GPs responding to the survey. Although this survey was conducted at the national level, the challenges it reveals are relevant to many European healthcare systems that are grappling with aging populations and increasing polypharmacy. Countries such as the UK and Slovenia have already structured deprescribing services in primary care. In contrast, Italy—and potentially other countries—must make greater efforts to integrate these practices into their national health services, fostering interprofessional collaboration and implementing consistent, structured educational pathways to support appropriate deprescribing.

The importance attributed to deprescribing by GPs in this survey, along with the reasons for its implementation [37]—first and foremost to maintain an acceptable risk/benefit profile of a therapy [38]—appears not to suggest a lack of awareness, but rather a practical challenge in implementing the process. The knowledge and understanding of daily practices and the reasons why deprescribing is applied sporadically are fundamental for designing appropriate improvement strategies.

Reluctance to modify a decision made by another clinician is one of the main factors indicated as limitations to deprescribing implementation, which was independent of GPs’ clinical experience. In another survey conducted in 2018 in Italy [39], nearly 70% of the GPs reported to feel confident with deprescribing; however, almost half of them expressed doubts regarding deprescribing when medication was initially prescribed by a colleague (45%). This barrier was reported also in other studies conducted in different countries [40, 41]. Indeed, in many healthcare systems, patients are seen by multiple specialists, such as cardiologists, endocrinologists, or neurologists, in addition to their GP. Each specialist may prescribe medications based on their specific area of expertise, often without full knowledge of the patient’s complete therapeutic regimen. There is undoubtedly a need for a coordinated approach, as well as for tools facilitating communication between different healthcare professionals. This is essential to ensure that patients feel supported by a synergistic system rather than by isolated practitioners who might make conflicting decisions. Beyond these needs, it is essential for deprescribing to be integrated into a broader healthcare framework and entrusted to designated healthcare professionals who serve as a point of reference. Nevertheless, GPs are uniquely positioned to implement deprescribing effectively due to their central role in coordinating patient care and their comprehensive understanding of the patient’s clinical and family history, comorbidities, and overall treatment plan. GPs oversee the patient’s health in its entirety, allowing them to identify unnecessary, redundant, or potentially harmful medications. Their long-term relationship with patients fosters trust and open communication, which are essential for discussing benefits and potential risks of deprescribing. Furthermore, GPs are well-placed to monitor the outcomes of medication withdrawal and make timely adjustments, ensuring patient safety and adherence to the revised treatment plan. Receiving formal endorsement from health authorities—along with transparent and structured communication between professionals —could help strengthen GPs’ role in deprescribing, improving their confidence while ensuring patient safety. Besides, another potentially useful strategy is the introduction of support roles, such as that of the pharmacist. In Italy, medication review is primarily the responsibility of GPs and specialists, with pharmacists playing an increasingly significant, yet still limited, role. In other healthcare settings, pharmacists play a critical role in reviewing medications, particularly in patients with polypharmacy [14, 15, 42, 43]. While the survey did not directly explore GP–pharmacist collaboration in medication reviews, the growing integration of pharmacists into primary care could offer valuable support for managing polypharmacy and promoting deprescribing. In Italy, clinical pharmacy is still developing and varies across regions, but closer collaboration between GPs and pharmacists could help optimize therapy and improve adherence to deprescribing practices.

Moreover, the same abovementioned response highlights the importance of targeted support interventions. Notably, GPs’ role in deprescribing is not without challenges. Based on the overall responses from this survey and the key findings from similar studies [18, 38, 44], we can identify three main areas that should serve as objectives for targeted interventions to support deprescribing practices. First, the development and dissemination of specific, shared, and formally recognized guidelines that can be easily applied to the most common cases and frequently used medications. While such documents do exist in the literature [45, 46], it is essential for scientific societies and health authorities to actively promote their adoption in clinical practice. Second, targeted training is needed to increase clinicians’ confidence in reducing or discontinuing medications and to equip them to handle complex situations [47]. This training should include continuing education for practicing clinicians and should also be integrated into general practice specialization programs, ensuring that even younger GPs—who often report to be less confident with deprescribing—receive adequate training and support. The third critical aspect that must be addressed is that GPs often face time pressures during consultations or visits, which can limit their ability to engage in detailed discussions about deprescribing. This issue requires a reorganization of workload and resources, allowing GPs to dedicate enough time to patients who need regular medication reviews. With a view to optimizing resources, it would be helpful to implement tools that enable clinicians to quickly identify patients with the most urgent needs, thereby prioritizing interventions effectively [48, 49].

Finally, one last aspect that deserves consideration concerns patients and their caregivers, who are often the first to resist therapy discontinuation. Although the direct relationship built by GPs with their patients is essential, this communication could be supported by informational materials and awareness campaigns implemented by local and national health authorities. At this stage, the role of other professionals, such as nurses or community pharmacists, could effectively support GPs’ efforts [50, 51].

This survey offers valuable insights for further consideration. We found that the majority of GPs were willing to deprescribe one or more medications in oldest-old multimorbid patients with polypharmacy, as also showed in the study by Jungo et al. [44] and previously in the LESS study [52]. Most of the discussion and research surrounding deprescribing has focused on older adults, largely due to the prevalence of polypharmacy in this population [53]. This finding reflects the choice to focus primarily on individuals who are subjected to polypharmacy and are at higher risk of drug-related adverse effects. Although the approach is understandable in the context of an attempt to prioritize, it raises concern that insufficient attention is being given to younger adults. Identifying patients who would benefit the most from deprescribing should become the standard practice, rather than relying solely on age as a determining factor for targeting individuals. By reducing the use of inappropriate medications earlier in life, patients can avoid future negative outcomes, such as preventable adverse drug reactions [54].

The study by Jungo et al. [44], of over 1,700 GPs from 31 countries, found variation in deprescribing decisions across countries and based on GP characteristics, such as age, with older GPs being more likely to take deprescribing decisions. Other previous studies reported that GPs with greater clinical experience were more able to draw on their own clinical knowledge [55], which might explain why older and more experienced GPs in our sample were more likely to deprescribe [38]. These age-based variances were confirmed by the differences observed in the tendency to deprescribe specific drug classes. For example, deprescribing antidepressants requires a cautious approach to avoid withdrawal side effects, but most importantly, it requires significant psychological support, which benefits from a strong relationship and communication between GP and patient [56]. This may explain why more experienced clinician report greater confidence in deprescribing these medications. On the other hand, deprescribing PPIs is a more standardized process with more evidence and supporting algorithms available [57], which may make it easier also for younger GPs to implement. These observations are valuable for informing the design of targeted strategies for GPs at different levels of experience. Finally, the lack of a nationally standardized deprescribing training framework in Italy may explain why many GPs express the need for specially trained professionals, despite engaging in deprescribing themselves. This suggests a structural gap in the Italian NHS, where deprescribing would benefit from more formalized and regulated support, as also highlighted in previous research [39].

There are several limitations to this study that should be acknowledged. First, the voluntary nature of the survey and the method of distribution may have introduced selection bias, as those GPs who have a particular interest in deprescribing or those more engaged in professional networks may have been more likely to participate. Additionally, the survey was self-reported, which could lead to response bias, particularly in questions related to attitudes and confidence. Finally, the online format may have excluded some GPs who do not frequently engage with digital platforms or social media. However, we have also to acknowledge the elevated number of respondents achieved by our survey. Moreover, including both early- to mid-career practitioners, who may be more familiar with recent advancements and contemporary guidelines, and more experienced GPs, who contribute with extensive practical, long-term experience allowed for an exploration of how attitudes toward deprescribing may vary between more recently trained GPs and those who have been practicing for decades. Future studies could benefit from combining quantitative surveys with qualitative interviews to provide a deeper understanding of the perspectives and experiences of GPs in deprescribing [58].

In conclusion, this survey aimed to explore GPs’ attitudes and confidence towards deprescribing, focusing on identifying barriers and understanding how deprescribing is integrated into clinical practice. The findings from this study shade light on the current state of deprescribing practices, and the specific challenges faced by GPs. To address these challenges, guidelines and education training on deprescribing approach are essential to equip GPs with the knowledge and confidence needed to reduce unnecessary medications safely. Furthermore, the integration of deprescribing into routine care, such as through enhanced medication review protocols, could support its role as a regular part of managing long-term conditions. By supporting GPs, healthcare systems can promote safer, more effective prescribing practices, reducing the risks associated with polypharmacy, improving patient outcomes, and decreasing costs for the NHS.

## Supplementary Information

Supplementary material.

## Data Availability

The raw data supporting the conclusions of this article will be made available by the authors without undue reservation.
