# Supplementary material for: Attitudes and confidence toward deprescribing: a survey among Italian general practitioners
Source: BMC Prim Care. 2025 Dec 23;26:403. doi: 10.1186/s12875-025-03042-2 (PMC12728995; doi:10.1186/s12875-025-03042-2)

**Supplementary Material**

**Attitudes and confidence toward deprescribing: a survey among Italian general practitioners**

Andrea Rossi^1,2^, Stefano Scotti^1,2^, Lara Perrella^3,4^, Federica Galimberti^2^, Elena Olmastroni^1,2^, Enrica Menditto^3,4^, Valentina Orlando^3,4^, Ilaria Ardoino^5^, Carlotta Franchi^5^, Manuela Casula^1,2^

1 Epidemiology and Preventive Pharmacology Service (SEFAP), Department of Pharmacological and Biomolecular Sciences, University of Milan, Milan, Italy

2 IRCCS MultiMedica, Sesto San Giovanni (Milan), Italy.

3 CIRFF, Center of Pharmacoeconomics and Drug Utilization Research, University of Naples Federico II, Naples, Italy.

4 Department of Pharmacy, University of Naples Federico II, Naples, Italy.

5 Laboratory of Pharmacoepidemiology and Human Nutrition, Department of Health Policy, Istituto di Ricerche Farmacologiche Mario Negri IRCCS, Milan, Italy.

**Corresponding Author**: Lara Perrella, CIRFF, Center of Pharmacoeconomics and Drug Utilization Research, Department of Pharmacy, University of Naples Federico II, Naples, Italy. E-mail: [lara-perrella@unina.it](mailto:lara-perrella@unina.it)

**SURVEY about DEPRESCRIBING**

**DEMOGRAPHIC DATA**

**GENDER**

- M
- F
- X, O, different, non-binary gender indication, or no indication

**YEARS OF WORK EXPERIENCE**

- <5 years
- 5-15 years
- 15-25 years
- >25 years

**GEOGRAPHICAL LOCATION**

- Northern Italy
- Central Italy
- Southern Italy and Islands

**SPECIALIZATION (if applicable)**

*Free text*

**DEPRESCRIBING – General Considerations**

**1. Based on your experience, which definition of deprescribing do you most agree with?**

- A review process consisting of dose reduction or discontinuation of therapy when the risk/benefit profile is no longer acceptable for the patient
- A process of discontinuing inappropriate therapy, supervised by a healthcare professional, with the aim of managing polypharmacy, improving outcomes, and reducing costs for the NHS
- A process aimed at safely discontinuing unnecessary or potentially harmful medications
- A review process of therapy in cases of ineffective treatment, adverse drug reactions, or when treatment goals have changed
- Other (specify)

**2. How relevant do you think deprescribing is in General Medicine? (1 = irrelevant; 5 = very relevant)**

| 1 | 2 | 3 | 4 | 5 |
| --- | --- | --- | --- | --- |

***(If response is ‘2-5’ to question 2)***

**2a In your opinion, what are the most important reasons for a doctor to implement deprescribing? (Multiple answers allowed)**

- Reduce the total number of medications taken by the patient
- Reduce costs for the NHS
- Prevent potential adverse drug reactions
- Align with updated therapeutic guidelines
- Reassess the risk/benefit ratio of therapy considering patient age and comorbidities
- Improve patient adherence
- Comply with regional/national deprescribing policies

**3. Which of the following factors should encourage deprescribing? (Multiple answers allowed)**

- Comorbid conditions or polypharmacy in the patient
- Side effects caused by chronic drug use
- Pathophysiological changes that could interfere with drug metabolism
- Advanced patient age
- Patient's socioeconomic condition
- Patient's desire to discontinue therapy
- Previous successful discontinuation of drug therapy

**4. Do you agree that General Practitioners (GPs) should be responsible for deprescribing?**

- Yes, it should be exclusively GPs’ responsibility
- Yes, but more time and resources are needed
- Yes, while evaluating whether to act personally or refer the patient to a specialist
- No, GPs could support specially trained physicians formally designated for this activity
- No, it should be exclusively managed by trained physicians designated for deprescribing

**5. Which of the following tools could facilitate deprescribing? (Multiple answers allowed)**

- Specific training on deprescribing
- Development/revision of accessible deprescribing guidelines
- Availability of criteria to identify potentially inappropriate medications
- Use of Decision Support Software (e.g., Micromedex, MSD Manual, Farmadati, INTERcheck, Codifa, etc.)
- Consultation service provided by trained professionals (e.g., a clinical pharmacologist)

**DEPRESCRIBING – Your Experience**

**6. How often do you implement deprescribing? (1 = never; 5 = very often)**

| 1 | 2 | 3 | 4 | 5 |
| --- | --- | --- | --- | --- |

***(If response is ‘1’ to question 6)***

**6a How applicable do you think deprescribing would be in your routine practice, considering time constraints and patient volume? (1 = not at all; 5 = very much)**

| 1 | 2 | 3 | 4 | 5 |
| --- | --- | --- | --- | --- |

**6b What are the reasons you do not implement deprescribing? (Multiple answers allowed)**

- Lack of time to review the patient's full clinical profile
- The therapy was not prescribed by myself
- Concern about adverse events and/or symptom recurrence
- Fear of resistance from the patient/family/caregiver
- Lack of useful guidelines for deprescribing
- Lack of literature on risk/benefit after deprescribing
- Insufficient experience in deprescribing
- Uncertainty about being sufficiently updated on the topic
- Other (specify)

***(If response is ‘2-5’ to question 6)***

**6a In which age group do you most frequently implement deprescribing?**

- Adults (≤64 years)
- Elderly (65-79 years)
- Very elderly (≥80 years)

**6b Which approach do you use to implement deprescribing? (Multiple answers allowed)**

- Explicit criteria (such as Beers, START/STOPP, etc.)
- Personal experience and knowledge
- Clinical evaluation of the individual patient
- Specific guidelines or algorithms
- Other (specify)

**6c How do you collect patient feedback after deprescribing?**

- I schedule a follow-up visit or phone consultation within a set time
- I wait for the next visit or opportunity to interact with the patient
- I wait for the patient to provide feedback
- I do not collect feedback

**6d What obstacles or difficulties do you find in deprescribing? (Multiple answers allowed)**

- Lack of time to review the patient’s overall clinical status
- Need to intervene on therapies prescribed by other physicians
- Concern about adverse events or symptom recurrence
- Resistance from the patient/family/caregiver
- Lack of useful guidelines for deprescribing
- Lack of literature on risk/benefit after deprescribing
- Limited experience in deprescribing
- Difficulty staying sufficiently updated on deprescribing
- Other (specify)

**6e How often do you deprescribe the following medications? (1 = never; 5 = often):**

**Benzodiazepines**

| 1 | 2 | 3 | 4 | 5 |
| --- | --- | --- | --- | --- |

**Antidepressants**

| 1 | 2 | 3 | 4 | 5 |
| --- | --- | --- | --- | --- |

**Antihypertensives**

| 1 | 2 | 3 | 4 | 5 |
| --- | --- | --- | --- | --- |

**Statins**

| 1 | 2 | 3 | 4 | 5 |
| --- | --- | --- | --- | --- |

**Bisphosphonates**

| 1 | 2 | 3 | 4 | 5 |
| --- | --- | --- | --- | --- |

**Proton Pump Inhibitors (PPIs)**

| 1 | 2 | 3 | 4 | 5 |
| --- | --- | --- | --- | --- |

**DEPRESCRIBING – PROTON PUMP INHIBITORS**

***(If response is ‘2-5’ to question 6e on PPIs)***

**7. In which age group do you most frequently deprescribe PPIs?**

- Adults (≤64 years)
- Elderly (65-79 years)
- Very elderly (≥80 years)

**8. What were the most common indications/symptoms/conditions in your PPI-treated patients who underwent deprescribing? (Multiple answers allowed)**

- Mild to moderate esophagitis
- Gastroesophageal reflux disease (GERD)
- Peptic ulcer disease
- Treatment of H. pylori infection
- Barrett’s esophagus
- Chronic NSAID therapy with high bleeding risk
- Severe esophagitis
- Documented history of bleeding ulcer
- Other (specify)

**9. What were the main reasons for deprescribing a PPI? (Multiple answers allowed)**

- The patient did not meet the indications for PPI therapy
- The condition could be managed through lifestyle modifications
- Risk of interaction with other medications
- The therapy was initially appropriate but is no longer needed
- The patient experienced adverse drug reactions (ADRs)
- The risk of ADRs was excessive
- Inadequate therapeutic response

**10. How do you implement PPI deprescribing? (Multiple answers allowed)**

- Immediate discontinuation
- Dose reduction followed by discontinuation
- Dose reduction
- On-demand use

**11. What level of acceptance have you observed in patients for PPI deprescribing? (1 = not accepted; 5 = well accepted)**

| 1 | 2 | 3 | 4 | 5 |
| --- | --- | --- | --- | --- |

**12. How often have you observed recurrence of the following symptoms after PPI deprescribing? (1 = never; 5 = always)**

**Heartburn**

| 1 | 2 | 3 | 4 | 5 |
| --- | --- | --- | --- | --- |

**Dyspepsia**

| 1 | 2 | 3 | 4 | 5 |
| --- | --- | --- | --- | --- |

**Regurgitation**

| 1 | 2 | 3 | 4 | 5 |
| --- | --- | --- | --- | --- |

**Epigastric pain**

| 1 | 2 | 3 | 4 | 5 |
| --- | --- | --- | --- | --- |

**Loss of appetite**

| 1 | 2 | 3 | 4 | 5 |
| --- | --- | --- | --- | --- |

**Weight loss**

| 1 | 2 | 3 | 4 | 5 |
| --- | --- | --- | --- | --- |

**Supplementary Figure 1** Responses to the question “Why a doctor should implement deprescribing” (respondents could provide more than one answer) – Stratification by years of professional experience


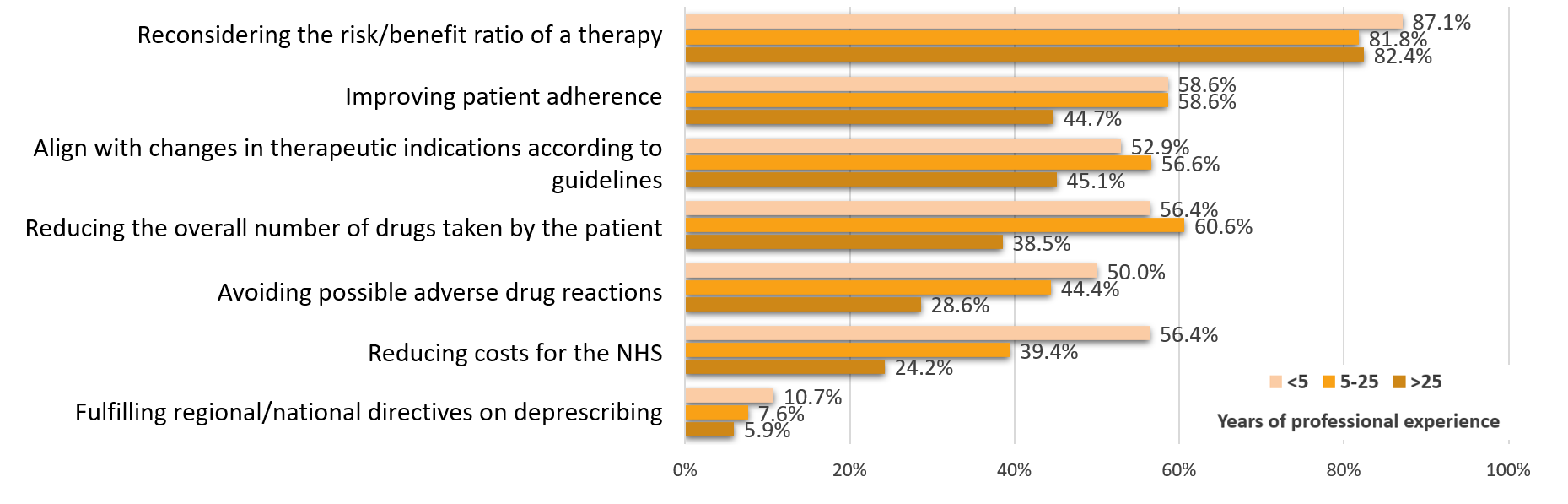


**Supplementary Figure 2** Responses to the question “Do you agree with the statement that deprescribing should be in charge to the general practitioner?” (respondents could provide only one answer) – Stratification by years of professional experience


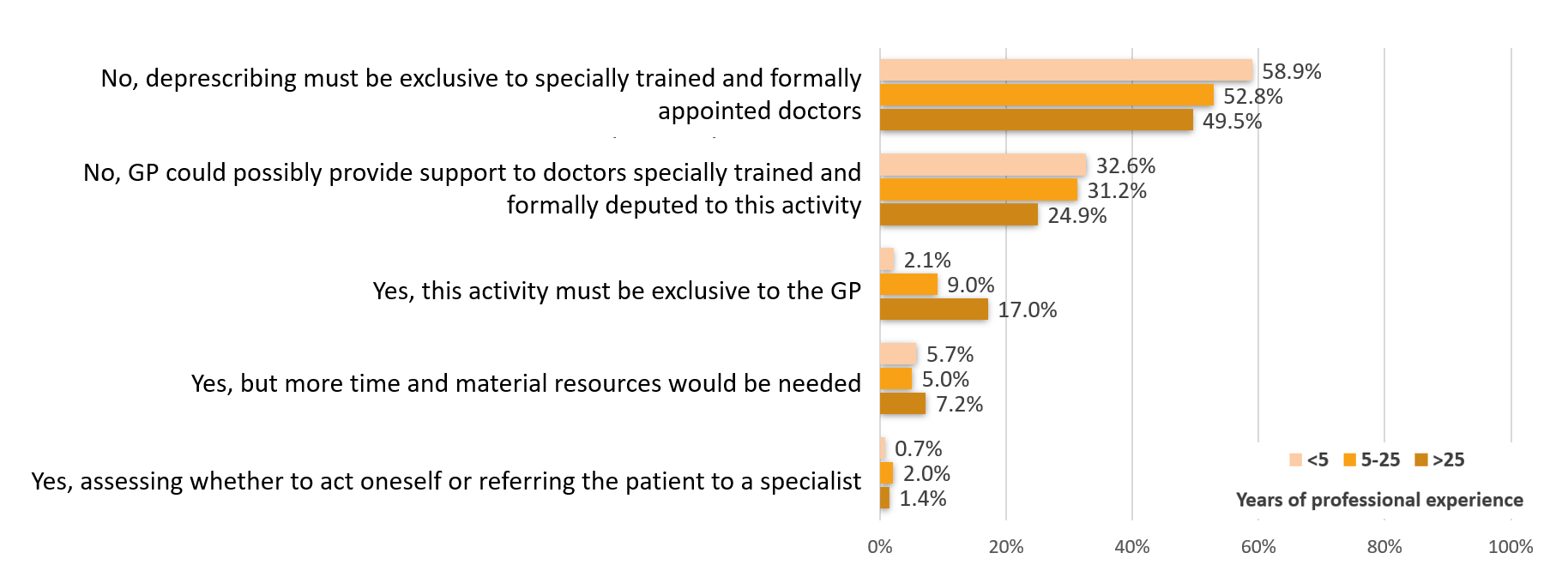


**Supplementary Figure 3** Responses to the question “What do you perceive as the main obstacles or challenges in the deprescribing process?” (respondents could provide more than one answer) – Stratification by years of professional experience

**
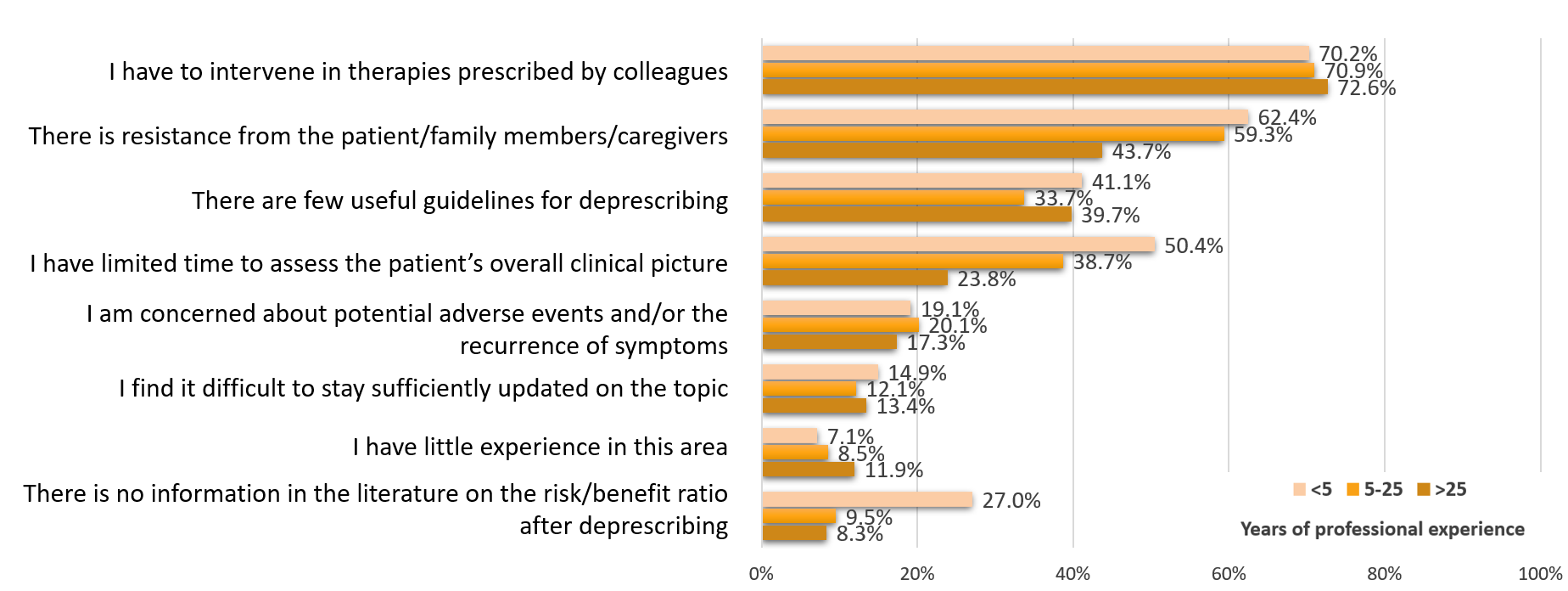
**

**Supplementary Figure 4** Responses to the question “Which of the following instruments could most facilitate deprescribing?” (respondents could provide more than one answer) – Stratification by years of professional experience


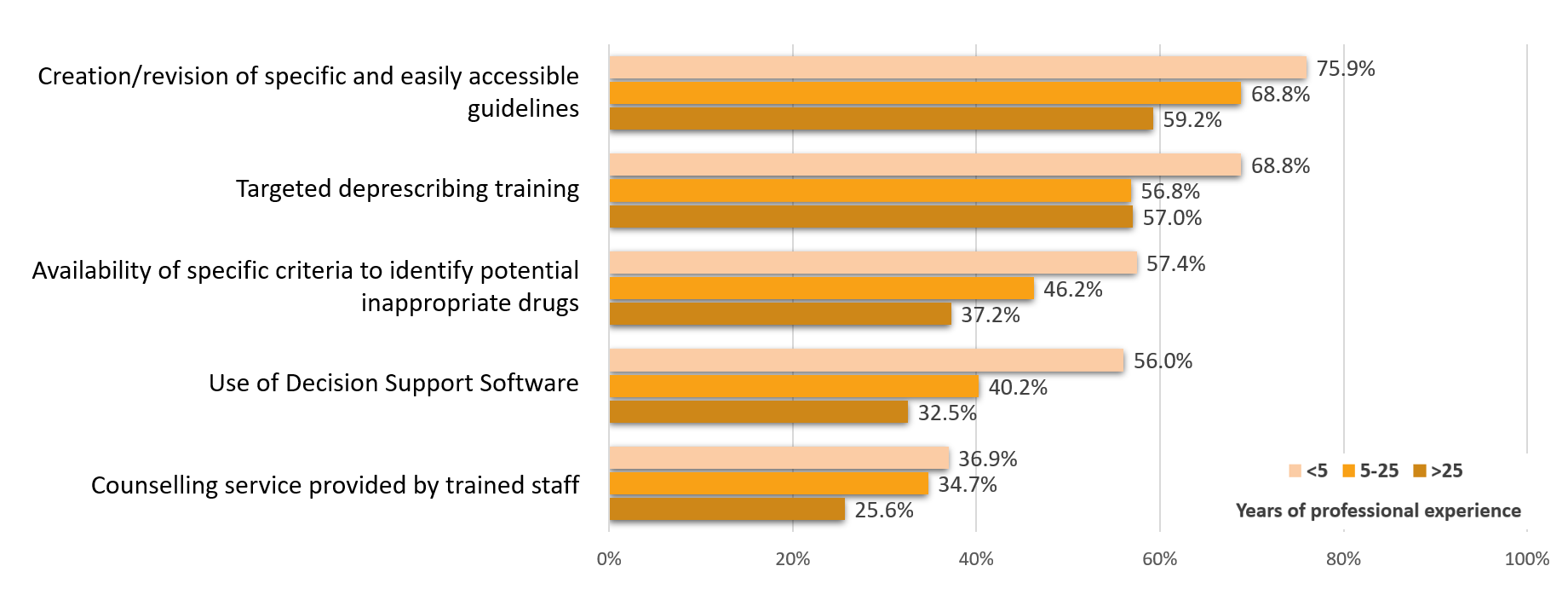

Supplement: Supplementary file 1 — Supplementary material. [file 12875_2025_3042_MOESM1_ESM.docx]
